# Dr. James B. Samo

**Published:** 1897-04

**Authors:** 


Dr. James B. Samo, of Buffalo, died at the Church Home in this city, where he had lived for a year or more, on Friday, March 12, 1897, aged 85 years. He was a native of Livingston county, New Jersey, but spent his early life at New York, where he pursued his medical studies with Dr. Hunter, and took his doctorate degree at the College of Physicians and Surgeons in 1832. He came to Buffalo in 1841 and joined the medical society of the county of Erie in 1844 ; was elected librarian of the society in 1852, holding the office until 1892 ; was president in 1862. He became a member of the Buffalo medical association upon its organisation in 1845, in which he held several offices and was elected president in 1864. Upon the organisation of the Buffalo general hospital he served for a time upon the medical staff and performed a similar duty at the hospital of the sisters of charity.

In his later years Dr. Samo lived in comparative retirement, though he retained his sprightliness of step and courtliness of manner until the very last. He will be long remembered as one of the solid members of the medical profession, who never lowered its standard and who stood for everything that goes to make up dignity and honor in its ranks. Elsewhere in this edition is recorded the action of his colleagues at a memorial meeting at which Dr. John Hauenstein presided, now the senior member of the profession in Buffalo, and who by singular coincidence joined the Medical Society of the County of Erie in the same year, 1844, as did Dr. Samo.

